# Association of Gln27Glu and Arg16Gly Polymorphisms in Beta2-Adrenergic Receptor Gene with Obesity Susceptibility: A Meta-Analysis

**DOI:** 10.1371/journal.pone.0100489

**Published:** 2014-06-24

**Authors:** Hongxiu Zhang, Jie Wu, Lipeng Yu

**Affiliations:** 1 Department of Obstetrics and Gynecology, The First Affiliated Hospital of Nanjing Medical University, Nanjing, China; 2 State Key Laboratory of Reproductive Medicine, Department of Obstetrics and Gynecology, The First Affiliated Hospital of Nanjing Medical University, Nanjing, China; 3 Department of Orthopaedics, The First Affiliated Hospital of Nanjing Medical University, Nanjing, China; Weill Cornell Medical College Qatar, Qatar

## Abstract

**Background:**

The beta2-adrenergic receptor (ADRB2) gene polymorphism has been implicated in susceptibility to obesity, but study results are still controversial.

**Objective:**

The present meta-analysis is performed to determine whether there are any associations between the Gln27Glu (rs1042714) or the Arg16Gly (rs1042713) polymorphisms in ADRB2 and obesity susceptibility.

**Methods:**

The PubMed (1950–2014), Embase (1974–2014), and China National Knowledge Infrastructure (CNKI, 1994–2014) databases were searched using the search terms (“Beta2-adrenergic receptor”, “β2-adrenergic receptor” or “ADRB2”), “polymorphism,” and “obesity”. Fixed- or random-effects pooled measures were determined on the bias of heterogeneity tests across studies. Publication bias was examined by Egger's test and the modified Begg's test.

**Results:**

Eighteen published articles were selected for meta-analysis. Overall analyses showed that rs1042714 (Gln27Glu) was associated with significantly increased obesity risk in the heterozygote model (Gln/Glu vs. Gln/Gln: OR: 1.16, 95% CI: 1.04–1.30, *I* ^2^ = 49%, P = 0.009) and the dominant model (Gln/Glu + Glu/Glu vs. Gln/Gln: OR: 1.2, 95% CI: 1.00–1.44, *I* ^2^ = 55%, P = 0.04), whereas no significant association was found in the other models for rs1042714. Also, no significant association was found between the rs1042713 (Arg16Gly) gene polymorphism and the risk of obesity in all genetic models. In addition, neither rs1042713 (Arg16Gly) nor rs1042714 (Gln27Glu) showed any significant association with obesity susceptibility when the population were stratified based on gender.

**Conclusion:**

Our meta-analysis revealed that the rs1042714 (Gln27Glu) polymorphism is associated with obesity susceptibility. However, our results do not support an association between rs1042713 (Arg16Gly) polymorphisms and obesity in the populations investigated. This conclusion warrants confirmation by more case-control and cohort studies.

## Introduction

Compelling evidence demonstrates that both obesity and other related traits have a significant genetic component [1], and that these phenotypes result from an interaction between the genetic background and environmental factors [2]. The beta2-adrenergic receptor gene (ADRB2), as a lipolytic receptor in human fat cells, is associated with lipid mobilization. The most common single nucleotide polymorphism (SNP) occurs at codon 16 (Arg16Gly; rs1042713) and codon 27 (Gln27Glu; rs1042714). By altering the amino acid sequence in the extracellular N-terminus of the ADRB2, the rs1042713 and rs1042714 allele mutations are believed to alter ADRB2 function [3].

A number of polymorphisms have been well-studied in ADRB2 and obesity. However, individual reports regarding ADRB2 polymorphisms with obesity have produced inconsistent results. For example, a previous study including 4,193 Japanese subjects indicated that rs1042713 was not a major contributing factor for obesity in Japanese men [4]. However, another study [5] showed that beta2-adrenoceptor polymorphisms may contribute to the development of obesity through gene-environmental interactions. Large et al. [6] found that in Swedish women obesity was associated with rs1042714, but not with rs1042713. The conflicting results of such studies may be a result of statistical underpower from sample sizes that were too small to detect any relationship between ARDB2 and risk of obesity. Therefore, we performed a meta-analysis of all published case-control or cohort studies to clarify the association of ADRB2 polymorphism with obesity susceptibility.

## Materials and Methods

### Publication search and inclusion criteria and exclusion criteria

The first report of significance of ADRB2 was published in 1954 [7], therefore we selected the starting date of 1950 (and last search date of April 19, 2014) for the article search in Pubmed, Embase, and the China National Knowledge Infrastructure. The search terms used were: “Beta2-adrenergic receptor”, “β2-adrenergic receptor”, “ADRB2”, “polymorphism,” and “obesity”. No language restrictions were imposed. For articles with overlapping data, we selected the publication with the most extensive data available.

To be included in the meta-analysis, the identified articles had to meet all the following criteria: a) evaluation of Gln27Glu (rs1042714) or Arg16Gly (rs1042713) polymorphism and obesity, b) inclusion of quantitative information on the estimated risk of ADRB2 Gln27Glu (rs1042714) and/or Arg16Gly (rs1042713) polymorphism for obesity, and c) inclusion of complete information about all genotype frequencies, d) used a case control or cohort or cross sectional design, randomization or blinding is not necessary; The exclusion criteria were as follows: a) papers not related to ARDB2 polymorphism and obesity research, b) review articles, commentaries, or unpublished reports, c) papers without usable data, and d) duplicate publications.

### Data extraction and quality assessment

We followed the Meta-analysis Of Observational Studies in Epidemiology (MOOSE) guidelines for reporting meta-analysis of observational studies [8]. The following items from each individual study were extracted: the name of the first author, year of publication, number of patients, gender, country, ethnicity, body mass index (BMI) cut point, sample size of obesity and control groups. The first stage was a review of titles and/or abstracts for all identified citations, followed by a second review stage of full text publications. Two reviewers (HXZ and LPY) independently assessed the eligibility of studies, and the third investigator (JW) arbitrated any disagreements by discussion and consensus. If allele frequencies were not provided, they were calculated from the corresponding genotype distributions. For information not available in the published paper, relevant data was obtained by contacting the corresponding authors. Two reviewers (HXZ and LPY) also assessed independently rated the methodological quality of every included study by the “Newcastle-Ottawa Quality Assessment Scale” (NOS)[9]. This scale contains nine items (1 point for each) in three parts: selection (four items), comparability (two items) and exposure (three items).

Some authors provided data only on subjects of one gender, some authors gave information on subjects of each gender, while others failed to report gender at all (gender not identified). The latter studies were included only in the group of both genders combined. Then we calculated the Hardy-Weinberg equilibrium for every study, both in the main group and in the gender-based subgroups. Eventually, 18 publications were enrolled in the main analyses, including 15 case control studies, one random study, one cross sectional study and one cohort study. Using these genotype comparisons, we pooled together the populations of both genders from all studies, and performed gender-based subgroup analyses that included all suitable studies. Nine publications were included for the ADRB2 Arg16Gly (rs1042713) gender-based groups and 17 publications were enrolled for the Gln27Glu (rs1042714) gender-based groups.

### Statistical analysis

Summary statistics were estimated in Review Manager 5.1 software (RevMan 5.1, Copenhagen: The Nordic Cochrane Center, The Cochrane Collaboration, 2011). The association between the rs1042714 gene polymorphism and obesity was compared by the odds ratio (OR) with its 95% confidence intervals (CIs). The statistical significance of the summary OR was determined with the Z-test. Five comparisons were performed between the two groups: frequency of allele (Gln vs. Glu), heterozygote comparison (Gln/Glu vs. Gln/Gln), homozygote comparison (Glu/Glu vs. Gln/Gln), dominant model (Gln/Glu + Glu/Glu vs. Gln/Gln) and recessive model (Glu/Glu vs. Gln/Gln + Gln/Glu) of ADRB2 Gln27Glu. For the rs1042713 (Arg16Gly) polymorphism, we used the same strategy, by replacing Glu27 with Arg16. Five comparisons were performed between two groups: frequency of allele (Arg vs. Gly), heterozygote (Arg/Gly vs. Arg/Arg), homozygote (Gly/Gly vs. Arg/Arg), dominant model (Arg/Gly + Gly/Gly vs. Arg/Arg) and recessive model (Gly/Gly vs. Arg/Arg + Arg/Gly) of ADRB2 Arg16Gly.

In consideration of the possibility of heterogeneity among the studies, a statistical test for heterogeneity was examined by the Chi-square-based Q-test, and the significance was fixed at the level P<0.05. The inconsistency index *I* ^2^ was also calculated to evaluate the variation caused by the heterogeneity. A high value of *I* ^2^ indicated a higher probability of the existence of heterogeneity. A random-effects model (DerSimonian and Laird method) was used if substantial heterogeneity was detected (Q-statistic: P<0.10; *I* ^2^>50%). Otherwise, a fixed-effect model was applied in the absence of between-study heterogeneity (Q-statistic: P>0.10; *I* ^2^< 50%). The significance of the pooled OR was determined by the Z-test, and the significance was set at P<0.05. Fisher's exact test was used to assess the Hardy-Weinberg equilibrium, and the significance was set at P<0.05. Potential publication bias was estimated using a funnel plot. Egger's linear regression test was used to evaluate the funnel plot asymmetry on the natural logarithmic scale of the OR (P<0.05 was statistically significant). We also further investigated the rs1042714 and rs1042713 gene polymorphism with obesity stratifying the population based on gender.

## Results

### Study characteristics

**Figure 1:**
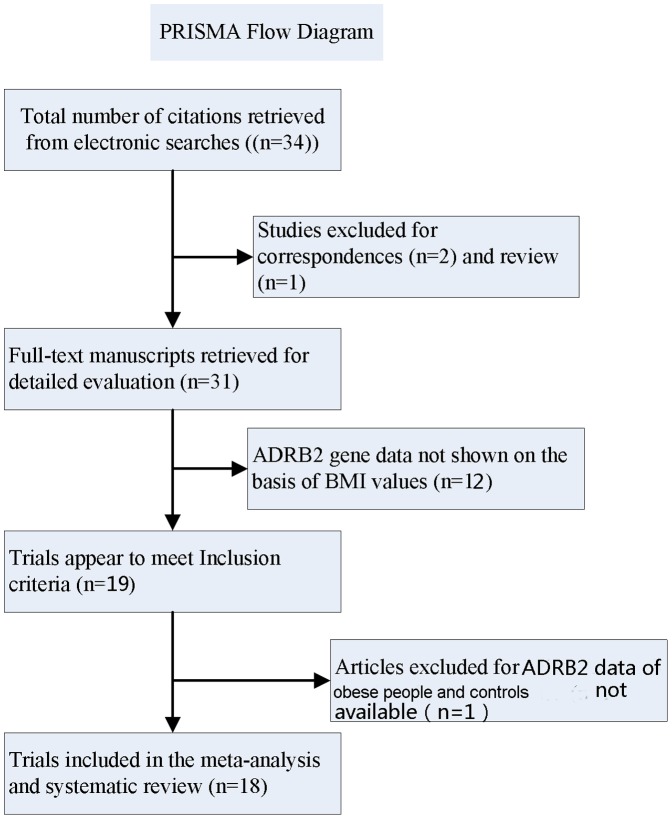
Flow diagram of articles selection process for ADRB2 gene polymorphism and obesity risk.

According to the search strategy, 34 published articles were identified for potential inclusion with full text obtained for ADRB2 polymorphism and obesity. Three articles were excluded because two papers were correspondences [10], [11] and another because it was a review [12]. 12 studies were excluded because groups were not divided by BMI [6], [13], [14], [15], [16], [17], [18], [19], [20], [21], [22], [23] and one paper was excluded due to the fact that data correlating ADRB2 with obesity were not available[24]. Thus, 18 studies [4], [6], [25], [26], [27], [28], [29], [30], [31], [32], [33], [34], [35], [36], [37], [38], [39], [40] met our inclusion criteria. Of these, 17 papers involving 9,995 subjects genotyped at rs1042714 (all except Angeli et al. [39]) and 10 studies [4], [6], [25], [26], [28], [30], [31], [33], [39], [40] including 7,322 subjects genotyped at rs1042713 were included. A flow chart of study selection is shown in Fig. 1. The distribution of genotypes in all included studies was consistent with the Hardy-Weinberg equilibrium. The key characteristics of these articles are summarized in Table 1.

**Table 1:** Characteristics of studies ofADRB2 polymorphisms between obese people and controls included in the meta-analysis.

Article (rs1042714)	Year	total number of patients	Study type	Gender(males/females)	Country	Ethnicity	BMI cut point	Sample size		
								Obesity	Normal weight	
Large et al [6]	1997	140	Case control	0/140	Sweden	Swedish	27	82	58	
Echwald et al [34]	1998	205	Case control	205/0	Denmark	Danish	27	78	127	
Hellstrom et al [35]	1999	247	Case control	138/109	Sweden	Swedish	27	125	122	
Kortner et al [36]	1999	442	Case control	184/258	Germany	German	40	243	199	
Mori et al [37]	1999	278	Case control	278/0	Japan	Japanese	26.4	61	217	
Ishiyama-Shigemoto et al [33]	1999	508	Case control	344/164	Japan	Japanese	27	108	400	
Oberkofler[31]	2000	399	Case control	0/399	Austria	Austrian	27	183	216	
Meirhaeghe et al [32]	2000	836	Random study	419/417	France	French	30	119	717	
Iwamoto et al [28]	2001	251	Case control	251/0	Japan	Japanese	25	151	100	
Kim et al [30]	2002	195	Cohort study	101/94	Korea	Korean	27	108	87	
Martinez et al [38]	2003	313	Case control	61/252	Spain	Spanish	30	159	154	
González Sánchez et al [27]	2003	666	cross sectional study	319/347	Spain	Spanish	30	186	477	
Malczewska-Malec[29	2003	38	Case control	38/0	Poland	Polish	30	22	16	
Masuo et al [26]	2006	329	Case control	329/0	Japan	Japanese	25	123	206	
Wu et al [40]	2009	396	Case control	223/173	China	Chinese	25	126	270	
Pereira et al [4]	2011	4193	Case control	2282/1911	Japan	Japanese	25	1200	2993	
Chou et al [25]	2012	559	Case control	275/284	Taiwan	Mixed	95th percentile	278	281	
Article (rs1042713)	Year	total number of patients	Study type	Gender(males/females)	Country	Ethnicity	BMI cut point	Sample size		
								Obesity		Normal weight
Large et al [6]	1997	140	Case control	0/140	Sweden	Swedish	27	82		58
Ishiyama-Shigemoto et al [33]	1999	508	Case control	344/164	Japan	Japanese	27	108		400
Oberkofler et al [31]	2000	399	Case control	0/399	Austria	Austrian	27	183		216
Iwamoto et al [28]	2001	251	Case control	251/0	Japan	Japanese	25	151		100
Kim et al [30]	2002	195	Cohort study	101/94	Korea	Korean	27	108		87
Masuo et al [26]	2006	329	Case control	329/0	Japan	Japanese	25	123		206
Wu et al [40]	2009	396	Case control	223/173	China	Chinese	25	126		270
Pereira et al [4]	2011	4193	Case control	2282/1911	Japan	Japanese	25	1200		2993
Angeli et al [39]	2011	361	Case control	Gender not identified	Brazil	African-derived Brazilian	25	140		221
Chou et al [25]	2012	559	Case control	275/284	Taiwan	Mixed	95th percentile	278		281

Abbreviations: BMI, body mass index; ADRB2, Beta 2-adrenergic receptor gene; Gln27Glu (rs1042714), at codon 27; Arg16Gly (rs1042713), at codon 16.

#### Methodological quality of 18 studies including our meta-analysis

Overall, the methodological quality of the 18 studies was modest. In general, the mixture studies including 15 case control studies, one random study, one cross sectional study and one cohort study. The studies failed to protect against selection bias: the definition of obesity used for the study is not uniform. None of the studies used secure methods for ascertainment of exposure. The majority of the studies provided evidence on the reliability of methods for outcome assessment; however, only several studies explicitly stated that outcome assessment was blind to exposure status. Finally, only two publications [4], [39] including in our meta-analysis clearly declared that no conflict with interest, others did not mention that (TableS1). Methodological quality of 18 articles enrolled in our study presented in Table 2.

**Table 2:** Methodological quality of 18 articles enrolled in our study by the “Newcastle-Ottawa Quality Assessment Scale”.

included studies	Selection				Comparability		Exposure			Total Quality score
Author	Is the case definition adequate?	Representativeness of the cases	Selection of Controls	Definition of Controls	Comparability of cases and controls on the basis of age	Comparability of cases and controls on nondiabetic subjects	Ascertainment of exposure	Same method of ascertainment for cases and controls	Non-Response rate	
year										
Large 1997	*	*	*	*	*	*		*	*	8
Echwald 1998	*	*	*	*				*	*	6
Hellstrom 1999	*	*	*	*	*	*		*	*	8
Ishiyama-Shigemoto 1999	*	*	*	*		*		*	*	7
Kortner 1999	*	*	*	*				*	*	6
Mori 1999	*	*	*	*				*	*	6
Meirhaeghe 2000	*	*	*	*	*	*	*	*	*	9
Oberkofler 2000	*	*	*	*		*		*	*	7
Iwamoto 2001	*	*	*	*	*			*	*	7
Kim 2002 Subjects exclude sex ration,age,blood pressure,serum LDL,HDL,serum triglycerides	*	*	*	*	*	*		*	*	8
González Sánchez 2003	*	*	*	*	*			*	*	7
Malczewska-Malec 2003	*	*	*	*		*		*	*	7
Martinez 2003	*	*	*	*				*	*	6
Masuo 2006	*	*	*	*	*	*		*	*	8
Wu 2009	*	*	*	*	*			*	*	7
Angeli 2011	*	*	*	*	*	*	*	*	*	9
Pereira 2011	*	*	*	*	*	*	*	*	*	9
Chou 2012	*	*	*	*	*	*		*	*	8

### Meta-analysis

**Figure 2:**
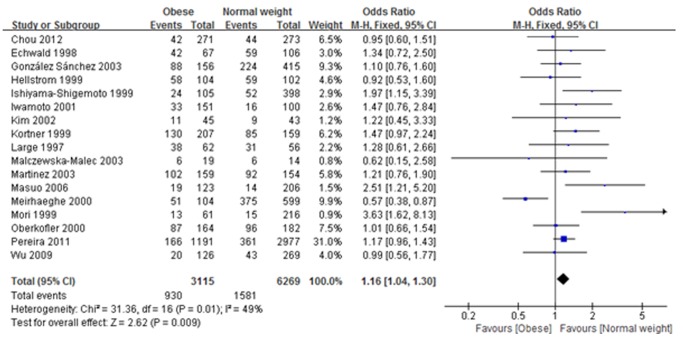
Association between rs1042714 (Gln27Glu) gene polymorphism and obesity risk under heterozygote model. (Gln/Glu vs. Gln/Gln: OR: 1.16, 95% CI: 1.04–1.30, *I* ^2^ = 49%, P = 0.009).

**Figure 3:**
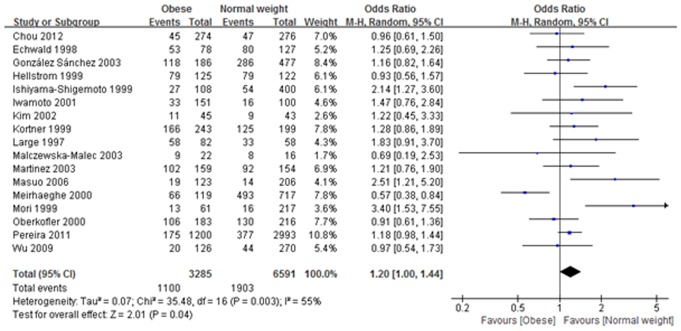
Association between rs1042714 (Gln27Glu) gene polymorphism and obesity risk under dominant model. (Gln/Glu + Glu/Glu vs. Gln/Gln: OR: 1.2, 95% CI: 1.00–1.44, *I* ^2^ = 55%, P = 0.04).

In the meta-analysis, 17 studies involved the rs1042714 (Gln27Glu) gene polymorphism, and 10 studies met our criteria for the rs1042713 (Arg16Gly) polymorphism. The frequency of occurrence of ADRB2 Gln27Glu/Arg16Gly allele in the population and their distribution among various populations are presented in table 3. A random-effects model was used if substantial heterogeneity was detected (Q-statistic: P<0.10; *I* ^2^>50%) or a fixed-effect model was applied in the absence of between-study heterogeneity (Q-statistic: P>0.10; *I* ^2^<50%). Meta-analysis of rs1042714 (Gln27Glu) and rs1042713 (Arg16Gly) polymorphism on risk of obesity are shown in Table 4 and Table 5, respectively. As shown in Table 4 and Fig. 2, in the analysis of the rs1042714 (Gln27Glu) gene polymorphism, the heterozygote model in our current study exhibited a significant difference (Gln/Glu vs. Gln/Gln: OR: 1.16, 95% CI: 1.04–1.30, *I* ^2^ = 49%, P = 0.009), indicating that risk of developing obesity with Gln/Glu heterozygotes was 1.16 times higher than those with Gln/Gln homozygotes. Additionally, the dominant model also exhibited a significant difference (Gln/Glu + Glu/Glu vs. Gln/Gln: OR: 1.2, 95% CI: 1.00–1.44, *I* ^2^ = 55%, P = 0.04), suggesting that risk of developing obesity with the Gln/Glu plus Glu/Glu genotype was 1.2 times higher than with the Gln/Gln homozygotes (Table 4 and Fig. 3). On the other hand, no significant correlation between obesity and Gln27Glu genetic variant in ADRB2 was found in the other three comparisons (Table 4). Our meta-analysis also showed that in all genetic models there was no significant association between Arg16Gly genetic variant in ADRB2 and the risk of obesity (Table 5).

**Table 3:** Distributions of ADRB2 Gln27/Glu and Arg16/Gly genotypes of eligible studies included in the meta-analysis.

year	Article (Gln27/Glu)	cases						controls				
		Gln/Gln	Gln/Glu		Glu/Glu	Gln27 allele frequency	Glu allele	Gln/Gln	Gln/Glu	Glu/Glu	Gln27 allele frequency	Glu allele
1997	Large et al [6]	24	38		20	86	78	25	31	2	81	35
1998	Echwald et al [34]	25	42		11	92	64	47	59	21	153	101
1999	Hellstrom et al [35]	46	58		21	150	100	43	59	20	145	99
1999	Kortner et al [36]	77	130		36	284	202	74	85	40	233	165
1999	Mori et al [37]	48	13		0	109	13	201	15	1	417	17
1999	Ishiyama-Shigemoto et al [33]	81	24		3	186	30	346	52	2	744	56
2000	Oberkofler[31]	77	87		19	241	125	86	96	34	268	164
2000	Meirhaeghe et al [32]	53	51		15	157	81	224	375	118	823	611
2001	Iwamoto et al [28]	118	33		0	269	33	84	16	0	184	16
2002	Kim et al [30]	34	11		0	79	11	34	9	0	77	9
2003	Martinez et al [38]	57	102		0	216	102	62	92	0	216	92
2003	González Sánchez et al [27]	68	88		30	224	148	191	224	62	606	348
2003	lczewska-Malec[29]	13	6		3	32	12	8	6	2	22	10
2006	Masuo et al [26]	104	19		0	227	19	192	14	0	398	14
2009	Wu et al [40]	106	20		0	232	20	226	43	1	495	45
2011	Pereira et al [4]	1025	166		9	2216	184	2616	361	16	5593	393
2012	Chou et al [25]	229	42		3	500	48	229	44	3	502	50
	Article (Arg16/Gly)	cases						Controls				
		Arg/Arg	Arg/Gly	Gly/Gly		Arg 16 allele	Gly allele	Arg/Arg	Arg/Gly	Gly/Gly	Arg 16 allele	Gly allele
1997	Large et al [6]	14	31	37		59	105	13	14	31	40	76
1999	shiyama-Shigemoto et al [33]	11	24	16		46	56	70	154	69	294	292
2000	Oberkofler [31]	37	83	63		157	209	36	102	78	174	258
2001	Iwamoto et al [28]	37	75	39		149	153	26	48	26	100	100
2002	Kim et al [30]	18	23	5		59	33	17	22	4	56	30
2006	Masuo et al [26]	27	62	34		116	130	77	88	41	242	170
2009	Wu et al [40]	36	72	18		144	108	82	138	50	302	238
2011	Pereira et al [4]	156	312	174		624	660	396	789	455	1581	1699
2011	Angeli et al [39]	26	77	37		129	151	45	107	69	197	245
2012	Chou et al [25]	47	62	28		156	118	46	60	28	152	116

ADRB2, Beta 2-adrenergic receptor gene; Gln27Glu (rs1042714), at codon 27; Arg16Gly (rs1042713), at codon 16.

**Table 4:** Meta-analysis of rs1042714 (Gln27Glu) polymorphism on risk of obesity.

Comparisons rs1042714 (Gln27Glu) polymorphism and obesity (17studies)	OR	95%CI	*I^2^*(%)	*P*
Allele frequency comparison (Gln vs. Glu)	0.86	0.74,1.01	61	0.06
Gender-based subgroup analysis with men	0.86	0.68,1.10	63	0.23
Gender-based subgroup analysis with women	0.86	0.67,1.09	67	0.21
Heterozygote comparison (Gln/Glu vs. Gln/Gln)	1.16	1.04,1.30	49	0.009
Gender-based subgroup analysis with men	1.22	0.90,1.65	62	0.21
Gender-based subgroup analysis with women	1.13	0.95,1.34	0	0.16
Homozygote comparison (Glu/Glu vs. Gln/Gln)	1.01	0.81,1.27	44	0.92
Gender-based subgroup analysis with men	0.9	0.63,1.29	25	0.58
Gender-based subgroup analysis with women	1.34	0.73,2.45	64	0.35
Dominant model (Gln/Glu + Glu/Glu vs. Gln/Gln)	1.2	1.00,1.44	55	0.04
Gender-based subgroup analysis with men	1.21	0.89,1.63	65	0.23
Gender-based subgroup analysis with women	1.15	0.97,1.35	39	0.1
Recessive model (Glu/Glu vs. Gln/Gln + Gln/Glu)	0.99	0.80,1.22	39	0.93
Gender-based subgroup analysis with men	0.92	0.66,1.30	0	0.65
Gender-based subgroup analysis with women	1.28	0.72,2.27	64	0.39

Abbreviations: OR, odds ratio; CI, confidence interval; *I* ^2^, Cochran's c–based Q-statistic test for assessing the heterogeneity (>50% indicates a substantial heterogeneity).

**Table 5:** Meta-analysis of rs1042713 (Arg16Gly) polymorphisms on risk of obesity.

Comparisons rs1042713 (Arg16Gly) polymorphism and obesity (10studies)	OR	95%CI	*I^2^*(%)	*P*
Allele frequency comparison (Arg vs. Gly)	1.02	0.95,1.10	25	0.52
Gender-based subgroup analysis with men	0.89	0.74,1.08	51	0.24
Gender-based subgroup analysis with women	1.1	0.99,1.23	27	0.08
Heterozygote comparison (Arg/Gly vs. Arg/Arg)	1.05	0.93,1.19	9	0.39
Gender-based subgroup analysis with men	1.1	0.91,1.31	27	0.32
Gender-based subgroup analysis with women	0.99	0.82,1.20	0	0.92
Homozygote comparison (Gly/Gly vs. Arg/Arg)	0.95	0.82,1.09	24	0.47
Gender-based subgroup analysis with men	1.1	0.90,1.36	44	0.34
Gender-based subgroup analysis with women	0.71	0.47,1.07	53	0.1
Dominant model (Arg/Gly + Gly/Gly vs. Arg/Arg)	1.02	0.91,1.14	15	0.74
Gender-based subgroup analysis with men	1.1	0.93,1.30	46	0.26
Gender-based subgroup analysis with women	0.92	0.77,1.11	0	0.39
Recessive model (Gly/Gly vs. Arg/Arg + Arg/Gly)	0.92	0.82,1.04	8	0.17
Gender-based subgroup analysis with men	1.04	0.88,1.23	0	0.61
Gender-based subgroup analysis with women	0.67	0.44,1.02	71	0.06

Abbreviations: OR, odds ratio; CI, confidence interval; *I* ^2^, Cochran's c–based Q-statistic test for assessing the heterogeneity (>50%indicates a substantial heterogeneity).

### Evaluation of publication bias

**Figure 4:**
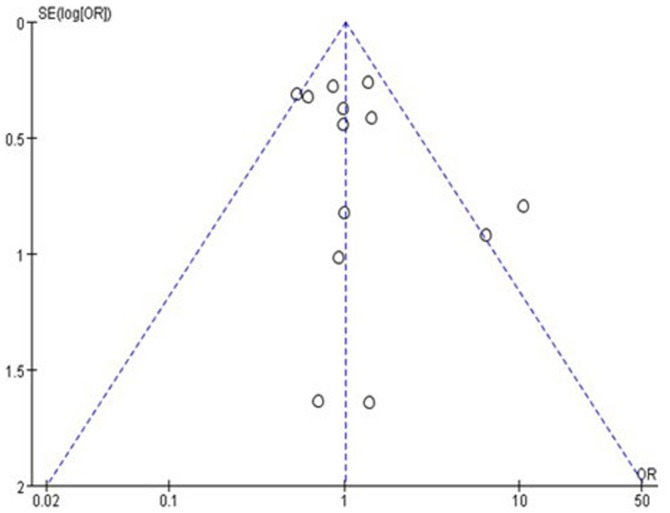
Funnel plot for rs1042714 (Gln27Glu) gene polymorphism on heterozygote Gln/Glu vs. Gln/Gln. The funnel plot showed no apparent evidence of publication bias.

Publication bias was assessed by the funnel plot and Egger's test. The funnel plot (heterozygote Gln/Glu vs. Gln/Gln) showed no apparent evidence of publication bias (Fig. 4). There was also no significant difference in Egger's test for the allelic genetic model, which suggested that the probability of publication bias was low in the present meta-analysis (t = 0.84, P = 0.424).

### Subgroup analyses by gender in the current study

Taking into account possible gender-specific roles in etiology [25], we conducted subgroup analyses by gender in the present study. After stratification for gender, no significant correlation of the rs1042714 (Gln27Glu) or rs1042713 (Arg16Gly) polymorphisms to obesity was observed in any of the genetic models (Table 4 and Table 5, respectively).

## Discussion

Several studies involved in ADRB2 polymorphisms and obesity have been published [4], [5], [6], [41]. However, published results are controversial. For example, Pereira et al. reported that Arg16Gly (rs1042713) was not a major contributing factor for obesity in Japanese men; however, it is believedto have a significant association with obesity in Japanese women [4]. Large et al. [6] reported thatADRB2 gene polymorphisms were markedly associated with obese Caucasian women. However, Echwald [34] and Oberkofler [31] found no association between ARDB2 and obesity. Specifically, a study performed by Oberkofler [31] concluded that the two polymorphisms of Gln27Glu (rs1042714) and the Arg16Gly (rs1042713) in the ARDB2 gene are not a major factor contributing to obesity in Austrian women. Moreover, Echwald et al. found the Glu27 polymorphism of ARDB2 gene is not associated with obesity in the population of Danish Caucasian men. However, Ehrenborg [42] found that individuals carrying the E27 allele and/or the G16 allele had significantly higher BMI, and that the E27 allele of the beta2-adrenoceptor gene is associated with slightly to moderately elevated BMI.

In 2007, Gjesting and colleagues [23] conducted a case-control study and meta-analysis examining 7,808 white people for eirany association between ADRB2 polymorphisms and obesity. In their study, genotype distribution of ADRB2 Gln27Glu and Arg16Gly was provided according to diabetic people and non-diabetic subjects. However the data of ADRB2 data according to obesity and controls were not available. Hence the article was excluded from our present meta-analysis. Their analysis provided the data of BMI stratified according to ADRB2 Gln27Glu and Arg16Gly genotype and they did not find significant correlation between these beta2-adrenergic receptor variants (both)and obesity. Furthermore, they did not find that the quantitative trait analyses showed any effect of the variants on obesity-related traits.

In 2008, Jalba and colleagues [43] performed a meta-analysis involving ADRB2 gene and obesity and conducted statistical analysis in three ways: the heterozygote comparison (Gln/Glu vs. Gln/Gln), the homozygote comparison (Glu/Glu vs. Gln/Gln) and the dominant model (Gln/Glu + Glu/Glu vs. Gln/Gln). Their results suggested that rs1042714 might be a significant risk factor for obesity in Asians, Pacific Islanders, and American Indians, but not in Europeans. Also, the report showed that obesity might not be associated with rs1042713 at all.

Since the association of ADRB2 polymorphisms with obesity is controversial, we conducted this meta-analysis based on all current available data on the relation between ADRB 2 polymorphism and obesity in 18 publications in order to clarify their relationship. Our findings suggest that there is a significant association between rs1042714 polymorphism of ADRB2 and obesity: OR = 0.86 for the allelic genetic model, OR = 1.2 for the dominant genetic model, OR = 0.99 for the recessive genetic model, OR = 1.01 for the homozygote genetic model, OR = 1.16 for the heterozygote genetic model.

In dominant comparison, the risk of developing obesity with Gln/Glu plus Glu/Glu genotype was 1.2 times higher than those with Gln/Gln homozygotes. However, our meta-analysis suggested no significant correlation of the rs1042714 (Gln27Glu) polymorphism to obesity in the other three comparisons. Also, no significant correlation to obesity was found for the rs1042713 polymorphism in all genetic models in the total population, as well as in gender-specific populations. This may be due to the divergence in genetic background. For example, the strength of the association between either rs1042713 or rs1042714 and obesity may be variable in different populations. Compared with the study of Jalba et al [43], we not only performed the following comparisons such as the heterozygote comparison (Gln/Glu vs. Gln/Gln), homozygote comparison (Glu/Glu vs. Gln/Gln) and dominant model (Gln/Glu + Glu/Glu vs. Gln/Gln), but we also conducted comparisons such as frequency of allele (Gln vs. Glu) and recessive model (Glu/Glu vs. Gln/Gln + Gln/Glu). In addition, we performed subgroup analyses by gender in the present study. Therefore, our results have stronger statistical power. Our meta-analysis suggest that Gln27Glu polymorphism of ADRB2 in heterozygote model showed a greater significance (p = 0.009) when compared to dominant model (p = 0.04). It may be a possible reflection of the linkage disequilibrium of genetic variability in codons 27 and gene-environment interaction in the etiology of obesity, since the mechanism of how Glu 27 can promote obesity is unknown at present. The results of our current meta-analysis support the conclusion that obesity susceptibility is associated with the Gln27Glu polymorphism of ADRB2 rather than the Arg16Gly polymorphism. This is different from previous results by Echwald [34] and Oberkofler [31] et al. Furthermore, our findings are inconsistent with the results of Gjesting [23]. No association of ADRB2 (both) with obesity risk was observed in our meta-analysis upon gender stratification. However, our results support the conclusion from Jalba [43].

Though our study provides the most comprehensive and up-to-date meta-analysis regarding the association between ADRB2 polymorphism with obesity, but also evaluates the methodological quality of 18 studies including our meta-analysis. Our work has some limitations. First, obesity is a complicated status involving complex interactions of genes, environment, and other factors, such as diet, lifestyle, diabetes mellitus, hypertension, total cholesterol, triglycerides, HDL and LDL. Though majority of publications included in our meta-analysis considered the factors described above such as diabetic factor, several articles did not consider the factors. See table 2. However, other susceptible factors were not able to be analyzed in the current study because insufficient data were provided from some of the original studies. Hence, misclassification bias is still possible. Moreover, only two studies [4], [39] explicitly stated that they have no conflict of interest, the others did not state whether they have conflict of interest or not. Therefore, possible conflicts of interests in studies enrolled in our meta-analyses may result in a bias.

Second, different BMI values were used as a cut-off in enrolling studies. Asian populations require a lower BMI to indicate that an individual is at the same risk as a European, and this variation can be explained by WHO expert consultations [44]. Out of our included studies, BMI of less than 30 was used in four out of five Asian studies at codon 27 and all seven Asian studies at codon 16. The specific details of variations in BMI cut point are shown in Table 1. It is not feasible to have the same BMI cut-off as obesity criteria across population from different geographical locations.

Third, our results were based on an unadjusted estimate, a more precise analysis should be performed adjusted by age, smoking, and other factors. Lack of the original data of the enrolled publications limit our further evaluation of potential interactions such as gene-gene, gene-environmental factors, which may affect obesity risk.

Lastly, although our funnel plot and Egger's test results showed no evidence of sample selection bias, it is still a remote possibility that such selection bias may have inadvertently occurred, and future meta-analysis studies may wish to recheck these conclusions as new research data continues to be published.

In summary, our results clarify that the overall conclusion of the literature to date indicates a significant association of Gln27Glu polymorphism with increased risk of obesity. Interestingly, increased risk of obesity is associated only with the Gln27Glu polymorphism of ADRB2, not the Arg16Gly polymorphism. This result sets the stage for future biochemical studies to investigate the mechanisms underlying this polymorphism-specific risk factor. Our conclusion also provides a basis to recognize patients at higher risk for obesity, allowing clinicians to more accurately create strategies for individualized therapy in obese patients.

## Supporting Information

Table S1 **Interest declaration of 18 studies included in the meta-analysis.**
(DOC)

Checklist S1 **PRISMA Checklist.**
(DOC)
