# Supplementary material for: Association of Gln27Glu and Arg16Gly Polymorphisms in Beta2-Adrenergic Receptor Gene with Obesity Susceptibility: A Meta-Analysis
Source: PLoS One. 2014 Jun 24;9(6):e100489. doi: 10.1371/journal.pone.0100489 (PMC4069060; doi:10.1371/journal.pone.0100489)
Supplement: Table S1 — Interest declaration of 18 studies included in the meta-analysis. (DOC) [file pone.0100489.s001.doc]

Table S1. Interest declaration of 18 studies included in the meta-analysis

| Author  year | conflict of interest |
| --- | --- |
| Large  1997 | Not mentioned |
| Echwald  1998 | Not mentioned |
| Hellstrom  1999 | Not mentioned |
| Ishiyama-Shigemoto  1999 | Not mentioned |
| Kortner 1999 | Not mentioned |
| Mori  1999 | Not mentioned |
| Meirhaeghe  2000 | Not mentioned |
| Oberkofler  2000 | Not mentioned |
| Iwamoto 2001 | Not mentioned |
| Kim 2002 | Not mentioned |
| González Sánchez 2003 | Partial support came from a grant by Elililly Co.,Spain |
| Malczewska-Malec  2003 | Not mentioned |
| Martinez 2003 | Not mentioned |
| Masuo 2006 | Not mentioned |
| Wu 2009 | Not mentioned |
| Angeli 2011 | no conflict of interest |
| Pereira 2011 | no conflict of interest |
| Chou 2012 | Not mentioned |
